# Neuromuscular electrical stimulation and dietary interventions to reduce oxidative stress in a secondary progressive multiple sclerosis patient leads to marked gains in function: a case report

**DOI:** 10.4076/1757-1626-2-7601

**Published:** 2009-08-10

**Authors:** David Reese, Ezzatolah T Shivapour, Terry L Wahls, Shauna D Dudley-Javoroski, Richard Shields

**Affiliations:** 1 Performance Therapies, PC, Ridgeway DriveCoralville, IowaUSA; 2 Department of Physical Therapy, University of Iowa Carver College of MedicineIowa City, Iowa, 52246USA; 3 Department of Neurology, University of Iowa Carver College of Medicine200 Hawkins Drive, Iowa City, Iowa, 52246USA; 4 Veterans Administration (VA), Iowa City VA Medical Center601 Highway 6 West, Iowa City, Iowa, 52246USA; 5 Center for Research in the Implementation of Innovative Strategies in Practice (CRIISP) VA HSR&D Center of ExcellenceIowa City VA Medical Center, 601 Highway 6 West, Iowa City, Iowa, 52246USA; 6 Division of General Medicine, Department of Internal Medicine, University of Iowa Carver College of Medicine200 Hawkins Drive, Iowa City, Iowa, 52246USA

## Abstract

Neuromuscular electrical stimulation has been used to aid musculoskeletal recovery. Excessive oxidative stress and excitoxicity are implicated in secondary progressive multiple sclerosis. A 52-year-old white female with SPMS had been scooter- and cane-dependent for 4 years. She requested and received a trial of neuromuscular electrical stimulation. Two months after initiating NMES the patient adopted several nutritional interventions to lower oxidative stress and excito-toxicity. During the first 2 months of neuromuscular electrical stimulation, the therapist observed modest gait improvements. Following the addition of nutritional interventions, more rapids gains in strength and endurance, including muscle groups not receiving neuromuscular electrical stimulation were observed by both the therapist and the patient. After 8 months of neuromuscular electrical stimulation (6 months of nutritional intervention) the patient’s function had improved sufficiently that she no longer used a scooter or cane and rode her bicycle routinely 8 miles, including hills.

## Introduction

The majority of those with relapsing remitting multiple sclerosis (MS) will go onto secondary progressive MS (SPMS) within 15 years of diagnosis. Neurodegeneration has been the presumed cause of the accumulating disability and loss of function [1].

Neuromuscular electrical stimulation (NMES) has been used to speed recovery after stroke [2]. Nutritional supplements and dietary interventions aimed at reducing oxidative stress and excito-toxicity are thought be benefit patients with MS [3,4]. Reduction of intracellular oxidative stress is associated with neuroprotection in experimental optic neuritis [5].

In this article we describe the use of physical therapy (PT), NMES-augmented exercises, and nutritional interventions in a patient with SPMS.

## Case presentation

A 52-year-old white female physician (tw) with secondary progressive multiple sclerosis (SPMS) was referred to physical therapy for evaluation and treatment of low back pain and gluteus pain. She had been diagnosed with MS in 2000. In 2003 her disease was reclassified as SPMS, at which time she started using a cane and an ankle foot orthotic (AFO) for left (L) foot drop. In 2004 she began using a scooter for fatigue. At presentation ambulation was limited to short distances (<20 yards). She sat semi-recumbent in a zero gravity chair for meals and desk work because of back fatigue. Stumbles and near falls were more likely to occur late in the day. Her MS medications included B complex vitamins, carnitine, lipoic acid, gabapentin, bupropion, baclofen, modafanil, mycophenolate, tolterodine, and minocycline. Bilateral glutei pain was reported. Low back pain (LBP) was centrally located and gradually progressed into the mid-thoracic region by evening. Pain was rated at 5 out of 10 for both.

PT notes indicated the presence of atrophy of her left lower extremity and glutei muscles. Her gait with a cane but without the AFO demonstrated a left foot slap during stance phase. Manual muscle tests revealed diffuse weakness of the core and lower extremities with left significantly weaker than right. Strength was rated 3 out of 5 for core muscles and left tibia anterioralis and 4 out of 5 for left hamstrings and quadriceps. Pain was attributed to abnormal posture and gait due to neuromuscular weakness. Patient and therapist goals were to increase strength and flexibility, and thereby improve safety and diminish pain. Prognosis was rated as fair.

### Intervention

After 3 months of usual PT care-stretching and core strengthening, utilizing both clinic sessions and a home exercise program (HEP)-the patient expressed a desire to try neuromuscular electrical stimulation (NMES). A test session was completed to confirm patient tolerance and capability of independent operation of the device. The patient was instructed to use a volitional muscle contraction along with the induced muscle contraction during NMES session (Table 1). The current, in milliamps, was increased within the patient tolerance for discomfort to attain a tetanic contraction in addition to her volitional contraction. Immediately following NMES the patient reported an enhanced sense of well-being following NMES. She also reported that unlike exercise, the NMES did not result in perceived muscle fatigue or generalized fatigue. A portable electrotherapy system 300 PV® manufactured by Empi was then acquired for home use. The patient was advised that an NMES time of 45 min per day was required to build muscle strength and 15 min per day was required for strength maintenance. She should use NMES on her abdominals and paraspinous muscle groups while completing her lumbar strengthening HEP. She could train additional muscle groups (using isometric volitional muscle contractions) as her schedule allowed.

**Table 1.** Electrotherapy device initial settings

Device	300 PV^®^	
	PP1 large muscle	Custom small muscle
Wave form	symmetrical	asymmetrical
Ramp on (seconds)	3	2
On time seconds)	12	5
Ramp off (seconds)	2	
Off time	20	5
Pulse rate (Hz)	35	50
Pulse width	300 µ	400 µ

Two months following initiation of NMES, the patient reported that she had made multiple nutritional interventions to reduce oxidative stress and excito-toxicity (based upon her review of the medical literature). Her typical daily intake included 600 grams of cruciferous vegetables, 300 grams of brightly colored fruits or vegetables, and 60 to 100 grams of meat, poultry or fish, but no milk, eggs, or gluten-containing grains. The patient also began the following supplements: 2 g each of glutathione, N acetyl-cysteine, and taurine daily, and lithium orotate 300 mg twice daily.

Five months following initiation of NMES, to facilitate increasing both the number of minutes and muscle groups receiving NMES, the patient acquired an eight-channel electrotherapy unit, the TDR68® manufactured by Tone-Amatic. The NMES protocol then consisted of 20 to 40 min of NMES to the upper and lower abdominals, paraspinous, both gluteus, and left hamstrings, quadriceps, hip flexors, and tibia anterioralis muscle groups each morning, in addition to four or more 30-min sessions of NMES, using her portable device, while at work.

To assess interventions used and functional gains accrued by the patient, we used patient reports of function and average minutes per muscle group of electrotherapy, and PT clinical notes. Because the patient had participated in the North American Research Committee on Multiple Sclerosis (NARCOMS) [6] patient registry since 2005, we reviewed self-reported disability scales from her responses to the NARCOMS quality of life questions before and during the intervention.

### Outcome

After 3 months of PT the patient’s back pain had diminished, but ambulation and sitting endurance were unchanged. She could do no more than 10 minutes of her HEP and still work due to fatigue limitations. However, after 2 weeks of NMES, the patient could complete 15 min of NMES-augmented HEP twice daily without difficulty. In addition the patient routinely completed another 60 to 90 min of NMES (while at work) of the abdominal, gluteus, and left anterior tibialis muscle groups. At 6 weeks the patient reported improved endurance for sitting and ambulation, although a cane was still required. The therapist observed gains in endurance and strength.

Within 2 weeks of initiating dietary interventions, the patient reported singing for the first time in 6 months. The therapist noted an increased rate of improvements in her strength and endurance, including muscles groups not receiving electrotherapy. The number of minutes and number of muscle groups were increased gradually. Four months following initiation of NMES the patient routinely did 30 minutes of NMES while completing her home exercise program each day and another 4 to 5 hours of NMES at much lower intensity through out the day while working or at home. Five months following initiation of NMES the patient stopped using her scooter, and 9 months after initiation of NMES the patient was able to bicycle 8 miles, including hills. One year following initiation of NMES and nutritional interventions the patient routinely rode her bicycle five miles to work.

The NARCOMS quality of life responses indicated gradual worsening of MS-related symptoms and disability scale prior to the intervention. Six weeks following initiation of NMES, improvement in overall symptoms and decreased fatigue were reported. Six months after initiation of NMES improvements were noted in overall MS symptoms, gait disability, and fatigue disability (Table 2).

**Table 2.** NARCOMS survey questions and patient responses

Date questions answered	11/23/2005	6/2/2006	11/28/2006	5/5/2007	12/12/2007^1^	4/30/2008^2^
Compare your overall MS symptoms now with what you experienced 6 months ago. Is your MS:	Worse	Worse	Worse	Worse	Somewhat Better	Much Better
Rate your MS symptoms overall	Moderate	Moderate	Moderate	Moderate	Minimal	None
Fatigue symptoms	Moderate	Severe	Total	Total	Moderate	Mild

^1^Six weeks after initiation of NMES.

^2^Six months after initiation of NMES and 4 months after nutritional intervention.

## Discussion

A 52-year-old white female with SPMS with 2 years of well-documented, gradual worsening of MS-related symptoms underwent NMES and dietary interventions to reduce oxidative stress and excito-toxicity. The patient experienced improved strength and endurance in response to the NMES. Following the dietary changes, the rate of improvements in function were more accelerated. To our knowledge this is the first published case report of the use of a NMES-augmented HEP and intensive nutritional support in a patient with progressive multiple sclerosis that resulted in significant reversal of disability.

The mechanisms by which NMES results in functional gains were likely due to changes both within the central nervous system (CNS) and the muscle. Physical activity has been associated with increases in nerve growth factor, brain-derived neurotrophic growth factor (BDNF), insulin-like growth factor, and glial growth factor [7,8].

Increased consumption of micronutrients appeared to have been synergistic with NMES. CNS response to circulating neurotrophins is dependent on the availability of intracellular adenosine triphosphate (ATP). Facilitating more effective mitochondrial bio-energetics with riboflavin, niacinamide, ubiquinone, and more antioxidants, could have facilitated enhanced responsiveness to neurotrophins, perhaps increasing dendritic sprouting and myelin generation.

Excessive neuronal excitation is present in experimental autoimmune encephalitis and patients with an acute MS relapse, primary progressive MS, and SPMS [9,10]. Blocking glutamate synthesis with taurine, glutathione, and N acetyl cysteine lowers excito-toxicity and has reversed axonal loss and disability in mice [11]. Antioxidants from food and nutritional supplements have been shown to inhibit T cell migration [12], block excito-toxicity [13], decrease oxidative stress [14] in both experimental autoimmune encephalitis and in multiple sclerosis patients.

## Conclusion

NMES and dietary manipulation aimed at reduction of oxidative stress and excito-toxicity in a patient with a 4-year history of SPMS was associated with large gains in patient function. This case suggests that the nutritional intervention and NMES were synergistic. Whether either NMES alone or nutrition alone would have yielded as many functional gains is unknown. Additional pilot studies are warranted to determine if these effects can be replicated. Careful patient selection criteria will be required to identify individuals capable of complying with these interventions because of the substantial time commitment to complete the NMES sessions, tolerance for discomfort without immediate benefit, and commitment to what may be substantial changes in dietary habits.

## Patient perspective

I had experienced gradual worsening of MS related symptoms since the diagnosis in 2000. It had been my expectation that continued worsening of my disability was inevitable. Following 18 months of neuromuscular electrical stimulation and intensive nutrition I can now bicycle 5 miles to work each day. I have noted when I am unable to eat 600 grams of cruciferous vegetables as when traveling, within 48 hours I experience subjective decline in energy and mental focus. When my electrical therapy device has had to be repaired and I have been without electrical stimulation for 48 hours I also experienced subjective decline in energy. It is my perception that two modalities (electrical therapy and antioxidant rich food and supplements) have additive, if not synergistic benefit to my recovery.

## Abbreviations

AFO: ankle foot orthotic
ATP: adenosine triphosphate
CNS: central nervous system
HEP: Home exercise program
LBP: Low back pain
NARCOMS: North American Research Committee on Multiple Sclerosis
NMES: neuromuscular electrical stimulation
PT: physical therapy
SPMS: secondary progressive multiple sclerosis
